# Apoplastic and intracellular plant sugars regulate developmental transitions in witches’ broom disease of cacao

**DOI:** 10.1093/jxb/eru485

**Published:** 2014-12-24

**Authors:** Joan Barau, Adriana Grandis, Vinicius Miessler de Andrade Carvalho, Gleidson Silva Teixeira, Gustavo Henrique Alcalá Zaparoli, Maria Carolina Scatolin do Rio, Johana Rincones, Marcos Silveira Buckeridge, Gonçalo Amarante Guimarães Pereira

**Affiliations:** ^1^Laboratório de Genômica e Expressão, Departamento de Genética, Evolução e Bioagentes, Instituto de Biologia, Universidade Estadual de Campinas-UNICAMP, CP 6109, Campinas-SP, CEP 13083-970, Brazil; ^2^Laboratório de Fisiologia Ecológica de Plantas, Departamento de Botânica, Instituto de Biociências, Universidade de São Paulo-USP, CP 11461, Rua do Matão 277, São Paulo-SP, CEP 05508-090, Brazil

**Keywords:** Autophagy, hemibiotrophic, *Moniliophthora perniciosa*, senescence, starvation, *Theobroma cacao.*

## Abstract

Witches’ broom disease (WBD) of cacao differs from other typical hemibiotrophic plant diseases by its unusually long biotrophic phase. Plant carbon sources have been proposed to regulate WBD developmental transitions; however, nothing is known about their availability at the plant–fungus interface, the apoplastic fluid of cacao. Data are provided supporting a role for the dynamics of soluble carbon in the apoplastic fluid in prompting the end of the biotrophic phase of infection. Carbon depletion and the consequent fungal sensing of starvation were identified as key signalling factors at the apoplast. *MpNEP2*, a fungal effector of host necrosis, was found to be up-regulated in an autophagic-like response to carbon starvation *in vitro.* In addition, the *in vivo* artificial manipulation of carbon availability in the apoplastic fluid considerably modulated both its expression and plant necrosis rate. Strikingly, infected cacao tissues accumulated intracellular hexoses, and showed stunted photosynthesis and the up-regulation of senescence markers immediately prior to the transition to the necrotrophic phase. These opposite findings of carbon depletion and accumulation in different host cell compartments are discussed within the frame of WBD development. A model is suggested to explain phase transition as a synergic outcome of fungal-related factors released upon sensing of extracellular carbon starvation, and an early senescence of infected tissues probably triggered by intracellular sugar accumulation.

## Highlight

Phytopathogens can co-opt plant primary metabolism to enhance pathogenesis and pathogen nutrition. In witches’ broom disease of cacao, sensing and modulation of compartmentalized carbon availability can also temporally regulate disease development.

## Introduction

Nutrition is a key component of the trade-offs involved in the evolution of parasitism, and this importance is highlighted by the remarkable diversity of strategies that parasites exploit in order to feed at their host’s expense (Newton *et al.*, 2010; Olson *et al.*, 2012). Among plant pathogenic fungi, three distinct classes have been described based on survival of host tissues during the course of nutrient acquisition: biotrophs, necrotrophs, and hemibiotrophs (Divon and Fluhr, 2007). Biotrophs establish longer term infections and derive nutrients exclusively from living tissues, while necrotrophs feed on dead tissues by benefiting from prematurely induced host cell death (Oliver and Ipcho, 2004). Hemibiotrophs present features of both strategies, initially deriving nutrients from living plant tissues, but also requiring host cell death to grow and complete their life cycle (Munch *et al.*, 2008). This short-term colonization of living tissues requires interaction-specific strategies for obtaining nutrients while completely avoiding or delaying host defence mechanisms (Mendgen and Hahn, 2002; Spanu *et al.*, 2010; Mukhtar *et al.*, 2011). This is a central theme in the evolution of the very specialized biotrophic and hemibiotrophic life styles. It probably provides the explanation for their generally narrower host range specificities, as well as the convergent evolution of complex feeding/effector delivery structures such as specialized intracellular hyphae and haustoria (Voegele and Mendgen, 2011).

A particularly well-studied model of hemibiotrophic fungi is the rice blast ascomycete *Magnaporthe oryzae* (Wilson and Talbot, 2009). In *M. oryzae*, starvation and autophagy are essential to trigger pathogenesis-related development (Kershaw and Talbot, 2009), and a tight co-ordination between nutrient acquisition and fungal growth avoids the early triggering of plant defences and temporally co-ordinates the transition to the necrotrophic phase (Fernandez and Wilson, 2012; Fernandez *et al.*, 2012). However, the complex intracellular nature of the interaction prevents a clearer understanding of the dynamics of nutrients during infection as well as its role, if any, in regulating disease development. In this regard, hemibiotrophs that have a strictly extracellular biotrophic phase may provide a somewhat simplified model for understanding how nutrient dynamics integrate into the hemibiotrophic life cycle.

Perhaps one of the most well-known hemibiotrophs with these characteristics is the tomato leaf mould *Cladosporium fulvum*. It enters leaves through stomata and cannot actively penetrate inside living host cells, growing restricted to the apoplast during the biotrophic phase (Perfect and Green, 2001). Carbon availability plays an important role in biotrophic development, as infected tissues experience a decrease of sucrose associated with invertase activity (Joosten *et al.*, 1990), and fungal proliferation occurs against the apoplastic sucrose gradient (Thomma *et al.*, 2005). Starvation-regulated genes are also induced during infection (Coleman *et al.*, 1997), and the importance of nutrient acquisition control is suggested by the decreased virulence of fungal mutants for the AREA transcription factor that act as a master regulator of preferable nitrogen source utilization in fungi (Thomma *et al.*, 2006; Bolton and Thomma, 2008). Remarkably, the disruption of AREA homologues in many phytopathogens appears to have less impact on the virulence of hemibiotrophs with a relatively short biotrophic phase (e.g. *M. oryzae* with 3–4 d). However, it strongly impairs pathogenicity in those that grow in the apoplast for an extended time (e.g. *C. fulvum* with 7–10 d; Divon and Fluhr, 2007), suggesting that tight regulation of nutrient acquisition and utilization is even more crucial for pathogens with long-lasting biotrophic phases.

A peculiar example of a hemibiotrophic phytopathogen with an unusually long biotrophic phase is the basidiomycete fungus, *Moniliophthora perniciosa*, the causal agent of witches’ broom disease (WBD) in cacao (Meinhardt *et al.*, 2008). Similarly to *C. fulvum*, it enters the host through stomata and thrives biotrophically at the apoplast (Frias *et al.*, 1991). However, this phase is greatly extended in WBD, as it can endure for >60 d (Calle *et al.*, 1982; Scarpari *et al.*, 2005). One remarkable feature of the hemibiotrophic cycle of WBD is the co-ordinated pleomorphic switch of *M. perniciosa* hyphae (Evans, 1980) in which the developmental stages of the typical sexual life cycle of Basidiomycetes parallels the symptomatic transitions of the infected cacao tissues. Biotrophic development is characterized by strictly extracellular monokaryotic hyphae (asexual mycelia) growing at the cacao apoplast. During this phase, infected stems are called ‘green brooms’ after their conspicuous diseased phenotype of loss of apical dominance, resulting in uncontrolled proliferation of abnormally swollen axillary shoots. The second, necrotrophic, stage occurs upon death of infected cacao tissues (and hence called ‘dry brooms’), and it is characterized by proliferative growth of the invasive dikaryotic hyphae (sexual mycelia) that are observed inside cacao cells (Purdy and Schmidt, 1996).

A role for a plant-derived carbon signal in the co-ordination of this transition has long been proposed (Evans and Bastos, 1980); however, identification of the precise compounds involved *in vivo* has proven difficult. *In vitro* studies demonstrated that glycerol can be used to maintain a biotrophic-like development (Meinhardt *et al.*, 2006), and the pharmacological blocking of the main respiratory chain sustained biotrophic-like growth in standard carbon-rich media (Thomazella *et al.*, 2012). In addition, different carbon sources mediate alterations in colony morphology and in the secretion of necrosis-inducing proteins *in vitro* (Alvim *et al.*, 2009), and evidence for a role of starvation and autophagy in WBD has been found both *in vitro* (da Hora Junior *et al.*, 2012) and *in vivo* (Pungartnik *et al.*, 2009). Previously a broad characterization of the biochemical alterations in whole infected tissues during WBD development was conducted (Scarpari *et al.*, 2005); however, the availability of soluble carbon and its signalling role in the main plant–fungus interface of WBD, namely the apoplast of cacao plants, remains largely unknown.

The general belief that the apoplast of plants is a relatively nutrient-poor environment (Wilson *et al.*, 2012) challenges the fact that *M. perniciosa* hyphae are capable of colonizing it for extended periods of time while inducing a remarkably strong set of phenotypic alterations in the host. In this study, the question of how the extracellular carbon availability is related to the long biotrophic phase of WBD in cacao is addressed, and its previously suggested role in the transition to the necrotrophic phase *in vivo* is explored. Efforts to build a detailed time course sampling of apoplastic soluble carbohydrates in healthy and diseased plants revealed remarkable dynamics which are strongly altered by WBD. This allowed the *in vivo* testing of the hypothesis that phase transition is prompted by carbon availability, followed by a stepwise collection of further physiological and molecular data depicting in unprecedented spatial–temporal detail the complex relationships of carbohydrate metabolism and WBD developmental transitions.

## Materials and methods

### Plant and fungal material

Experiments were performed using the strain FA553 of *M. perniciosa* (Mondego *et al.*, 2008), routinely maintained in MYEA medium (2% malt extract, 0.5% yeast extract, 2% agar; w/v) in the dark at 28 ºC. Spores were obtained as described by Pires *et al.* (2009) and Frias *et al.* (1995). *Theobroma cacao* (variety Catongo) half-sibling plants were cultivated from selected seeds obtained from fruits collected from the same mother tree. Plants were cultivated into 10cm^3^ pots of standard soil/vermiculite mixture (1:1, v/v) and watered daily with tap water in a greenhouse with controlled environment conditions (temperature of 25–30 ºC, photoperiod of 12h at an average of 3000 lux, and humidity >70%).

### Seedling inoculation and apoplastic fluid extraction

Forty-day-old seedlings had their apical meristem pruned for homogenous induction of lateral stem growth from dormant axillary buds. One bud per plant was selected for inoculation when bearing the first new leaf ranging from 3mm to 5mm in length. Any additional active axillary buds from the same plant were removed by pruning. Inoculation solutions and spores were handled according to Frias *et al.* (1995) with modifications: 10^6^ ml^–1^ spore solutions were pipetted directly into the young leaves from lateral buds. Plants were photographed and harvested for apoplastic fluid extraction during the same period of the day before the daily watering. Lateral stems were cut with a razor blade, the cut end was briefly washed with distilled water, and they were placed into an adapted Scholander pressure bomb (Ruan *et al.*, 1995) (Supplementary Fig. S1 available at *JXB* online). Each extraction was conducted using nitrogen to apply a pressure of 20 Bar for 5min into the sealed chamber while collecting apoplastic fluid from the air-exposed end of the stem. Samples were snap-frozen in liquid nitrogen and kept at –80 ºC for later chromatography of carbohydrates. The extracellular nature of the collected fluid was validated by glucose 6-phosphate isomerase activity according to Pirovani *et al.* (2008) and by comparison of amino acid profiles with whole leaf and stem extract (Supplementary Fig. S2).

### Chromatography of apoplastic fluid carbohydrates and invertase assays

Carbohydrates were analysed directly from collected apoplastic fluid using an ion exchange modular chromatography system (Metrohm, Herisau, Switzerland) consisting of an 818 IC pump, 830 IC Interface, and 817 Bioscan containing the column and the pulsed amperometric detector (PAD). Separations were conducted on a Metrosep Carb-250 column using degassed 0.1M NaOH as isocratic eluent in a flow rate of 1ml min^−1^ for 30min, and identified and quantified using standard curves built with standard stock dilutions (obtained from Sigma). Cell wall (cw-) and vacuolar (vc-) invertase assays were conducted on whole tissue extracts essentially as previously described (Roitsch *et al.*, 1995).

### Nucleic acid manipulations and gene expression analysis

*Moniliophthora perniciosa* DNA extractions were performed essentially as described previously (Mondego *et al.*, 2008). Fungal total RNA was isolated using the Plant RNeasy Mini kit (Qiagen). Total RNA from cacao was extracted from tissues pulverized in liquid nitrogen using a mortar and pestle (whole tissues) or a Mini-Beadbeater-96 (BioSpec Products, USA) with 1mm steel beads (leaf discs or cells). A 50mg aliquot of frozen tissue was homogenized by shaking in 2ml of a 1:1 (v/v) mixture of chloroform:extraction buffer [2% cetyl trimethylammonium bromide (CTAB); 1% sarkosyl; 2M NaCl; 0.2M sodium borate; 100mM TRIS-HCl, pH 8.0; 30mM EDTA; 5% β-mercaptoethanol; 2% poly(vinylpyrrolidinone) (PVP)]. Samples were spun at 25 000 *g* and 4 °C (Eppendorf 5417R) for 10min, the supernatant was re-extracted with 1/2vol. of chloroform, and the RNA was precipitated with 1/3vol. of 12M LiCl on ice for 4h. RNA was pelleted at 25 000 *g* and 4 °C for 30min, and rinsed with ice-cold 3M LiCl following another 10min spin. Typically 3–4 replicates from one individual point were each resuspended in 50 μl of diethylpyrocarbonate (DEPC)-treated water, pooled, and re-precipitated overnight by standard ethanol–sodium acetate precipitation. Fungal and plant RNA samples were assayed using a NanoDrop ND-1000 spectrophotometer (Thermo Fisher Scientific Inc.) followed by 1% formaldehyde–agarose denaturing gel. cDNA was synthesized from 2 μg of total RNA primed with random primers using the Superscript II Reverse Transcriptase kit (Invitrogen). All quantitative real-time PCRs (qRT-PCRs) were performed using 0.2 μM of each primer and SYBR Green Master Mix in a StepOnePlus system under recommended conditions and quality controls (Applied Biosystems). Expression levels for each treatment were calculated using the formula [2^–(Ct target–Ct internal reference)^]×1000 and either conveyed as normalized absolute levels as is, or as the fold increase obtained by dividing by the values obtained in appropriate controls when applicable. All primer sequences are listed in Supplementary Table S1 at *JXB* online.

### Photosynthesis, whole-leaf carbohydrate assays, and petiole infiltration assays

Photosynthesis was assayed using a portable system (LI-6400 XT, Li-Cor, Lincoln, NE, USA), equipped with a fluorescence chamber (LI-6400–40, Li-Cor). All assays were conducted at 28±1 °C and 40 ppm CO_2_. Maximal assimilation (*A*) and electron transport rate (ETR) were determined using 800mm photons m^2^ s^–1^ as the saturation point (Long and Bernacchi, 2003) with simultaneous assessment of control parameters according to Oxborough and Baker (1997). After measurements, all leaves were separately collected and snap-frozen in liquid nitrogen for later RNA extraction and carbohydrate analysis.

Soluble non-structural carbohydrates were extracted four times from 0.01g of powdered samples in 80% ethanol (80 °C) for 20min. Extracts were pooled and vacuum dried, re-suspended in 1ml of deionized water, and pigments were removed by the addition of 0.5ml of chloroform. Soluble sugars in the aqueous phase (glucose, fructose, and sucrose) were quantified against standards (Sigma) by high-performance anion exchange chromatography with pulsed amperometric detection (HPAEC/PAD) using a Dionex-DX500 system (Dionex Corporation, Sunnyvale, CA, USA) equipped with a CarboPac PA1 column. Chromatography was performed using 100mM NaOH as eluent, with a flow rate of 1ml min^–1^. Starch was assayed from the insoluble pellets as described (Smith and Zeeman, 2006).

Petiole feeding assays were conducted as described in Lin *et al.* (2011) with modifications: petioles from fully expanded leaves at the desired position were cut mid-length with a thin razor blade followed by a quick wash with distilled water and the rapid attachment of the infiltration apparatus containing the desired solutions. Infiltrations were most successfully conducted by applying a slight pressure through the use of rubber bands on the plunger of each syringe. Typically 75% of the infiltrations were successful based on traceable xylene cyanol dye migration, and all successfully infiltrated petioles remained functionally attached to the plants for at least 7 d. All solutions were prepared in distilled water.

### Plant carbon starvation treatments

Starvation by dark treatment was performed by enclosing whole plants in a dark compartment of the greenhouse for 1 week before RNA extraction. Cacao cell cultures were obtained from callus induced on cotyledon explants incubated for up to 60 d in callus-inducing media at 25 ºC and 120rpm in the dark (1× Murashige and Skoog salts; 1× Gamborg’s vitamins solution; 20g l^–1^ sucrose; 2mg l^–1^ 2,4-dichlorophenoxyacetic acid; 2mg l^–1^ indole butyric acid; 2mg l^–1^ indole acetic acid). Cells were maintained in liquid media with passages to fresh media every week. Starvation treatment was acheived by using culture medium without sucrose.

### Genetic manipulations and fungal autophagy monitoring

The polyubiquitin promoter, terminator, and the coding region of the autophagy-related gene 8 of *M. perniciosa* (*MpATG8*) were PCR amplified from FA553 strain genomic DNA, and *EGFP* (enhanced green fluorescent protein) and the hygromycin (*hph*) selection cassette (Supplementary Fig. S5 at *JXB* online) were amplified from the pBGgHg vector (Chen *et al.* 2000) using the primers listed in Supplementary Table S1. Purified products were assembled by fusion PCR cloning as described (Szewczyk *et al.*, 2006).

Fused cassettes were cloned in pGEM-T-easy (Promega) and confirmed by sequencing. The final vector for transformation of *M. perniciosa* was obtained by sequential digestion–ligation of the EGFP–MpATG8 expression cassette into the vector containing the hygromycin selection cassette using *Sac*I and *Sal*I restriction endonucleases. Transformation of *M. perniciosa* protoplasts followed the same procedures described by Lima *et al.* (2003), with the exception that transformed protoplasts were plated into 20ml of solid protoplast regeneration medium (0.5% peptone, 0.5% yeast extract, 0.3% glucose, 0.1% mannitol, 0.5% glycerol, 0.8% malt extract, 17% sucrose, and 1% agar) without selection for 3 d followed by the overlay of 5ml of semi-solid (0.7% agar) protoplast regeneration medium containing 1mg ml^–1^ hygromycin (for a final selective concentration of 200 μg ml^–1^ on the plate).

Confirmed transformants were cultivated into solid plates of 2% agar in tap water for 7 d and then transferred to defined solid medium according to Metzenberg (2004) modified by exchanging agar by agarose as the solidifying agent and either with or without carbon sources. Rapamycin treatments were conducted by overlaying 15-day-old colonies with 5ml of semi-solid (0.7% agarose) defined media containing 500ng ml^–1^ rapamycin. Hyphae for RNA extraction or microscopy were carefully scraped and collected from cracked open agarose layers after 12h of treatment. Slides containing live hyphae mounted in liquid medium were photographed by standard confocal laser microscopy (Carl Zeiss, LSM 510 Meta) and processed using LSM Image Browser (Carl Zeiss).

## Results

### A periodic carbon cycle in the apoplastic fluid of cacao

**Fig. 1.**
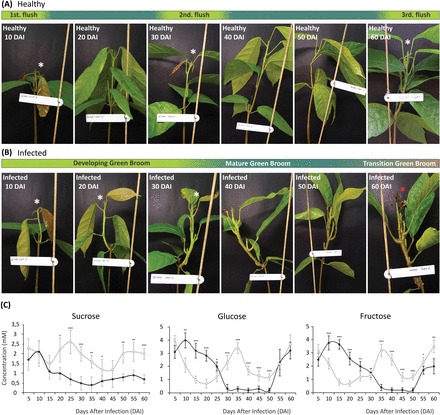
Development of WBD in cacao seedlings during the first 60 days after infection (DAI) of the time course experiment for assessing apoplastic fluid carbohydrate dynamics. (A) Representative pictures comparing the developmental stages of healthy plants (upper panel) and (B) infected plants (lower panel), highlighting the differences in the periods of apical meristem activity (white asterisks) and the first appearance of necrosis in infected plants (black asterisk). (C) Millimolar concentrations of sucrose, glucose, and fructose in the apoplastic fluid of healthy (open circles) and infected seedlings (filled circles). Data points represent the mean of four biological replicates with calculated standard deviations (unpaired Student’s *t*-test; *P<0.05; **P<0.005; ***P<0.0005). (This figure is available in colour at *JXB* online.)

Apoplastic fluid was isolated from healthy and infected stems using an adapted Scholander pressure bomb (Supplementary Figs S1, S2 at *JXB* online), and the soluble carbohydrate profile was determined using liquid chromatography. In order to obtain a view of the soluble carbon sources available to *M. perniciosa* in the apoplast during infection, quantitative analysis was performed in a time course experiment spanning the whole biotrophic and the initial transition to the necrotrophic phase of WBD in cacao seedlings. In healthy plants, concentrations of apoplastic carbohydrates followed an apparent cyclic pattern, successively alternating between higher and lower levels throughout the duration of the experiment. While the concentration of glucose and fructose peaked three times with very similar intervals, sucrose concentrations appeared to oscillate in an inverse manner (Fig. 1C; open circles). The observed pattern coincided with the developmental cycle of cacao seedlings as high levels of carbohydrates overlapped with periods of new leaf flushing and vegetative growth (Fig. 1A; Supplementary Fig. S3).

### WBD disrupts the carbon cycle in the apoplast of cacao

**Fig. 2.**
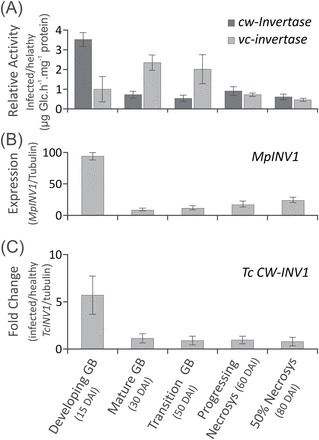
Whole-tissue invertase enzymes activities and gene expression measured at key developmental stages of WBD, spanning from green broom stages (GB) to early necrosis. (A) Cell wall (cw-), and vacuolar (vac-) invertase activities portrayed as fold increase in infected tissues. (B) Gene expression of a conserved fungal secreted (extracellular) invertase present in the *M. perniciosa* genome. (C) Gene expression of a conserved cacao cw-invertase present in the *T. cacao* genome. Data points represent the mean value of three biological replicates with calculated standard deviations.

Disease symptoms followed a similar developmental time frame to what was previously described when using seedlings as models for WBD development (Scarpari *et al.*, 2005). Infected cacao shoots lost the periodic growth pattern characteristic of healthy controls (Fig. 1B), and this loss was also reflected in the disruption of the pattern of carbohydrates in the apoplastic fluid (Fig. 1C; filled circles). Glucose and fructose levels were found to be systematically higher in the apoplastic fluid of infected plants in the first 25 d of WBD development (Fig. 1C), during the tissue-proliferative phase of the disease (Fig. 1B; Supplementary Fig. S3 at *JXB* online). The increase of glucose and fructose correlated with the decrease of sucrose levels, suggesting enhanced activity of invertases. Assays for cw- and vc-invertase activity revealed that cw-invertase is 3.5 times more active specifically in developing green brooms, showing little or no difference between healthy and infected tissues at later stages (Fig. 2A). Remarkably, vc-invertase is twice as active in infected tissues at the stage of mature and transition green brooms, with no difference at the beginning of infection or at later necrotic stages (Fig. 2A). In addition, gene orthologues of predicted cacao and *M. perniciosa* extracellular invertases appeared to be up-regulated in developing green brooms (Fig. 2B, C), indicating a possible cumulative role between fungal and plant enzymes in the maintenance of high apoplastic hexose levels from the breakdown of free sucrose.

The mature broom stage was phenotypically characterized by the cessation of growth of the infected tissues [40 days after infection (DAI); Fig. 1B; Supplementary Fig. S3 at *JXB* online]). In this stage, all apoplastic fluid carbohydrate concentrations reached the lowest levels (Fig. 1C; filled circles), indicating that the cacao apoplast is relatively depleted of soluble carbon at advanced stages of infection. Hexoses appear again at measurable levels only after 50 DAI (Fig. 1C; filled circles), together with the appearance and spreading of leaf necrosis (Fig. 1B; 50–60 DAI), but with no clear increase in either activity or gene expression of apoplastic invertases (Fig. 2A–C), probably reflecting the leakage of the intracellular content of dying cacao cells. These results indicate that infection leads first to an extent of apoplastic fluid hexose accumulation and then to the depletion of soluble carbohydrates before the transition to the necrotrophic phase. Based on the present findings of carbon depletion in the apoplastic fluid of mature brooms and on previous reports showing that fungal growth is strictly apoplastic up until much later in WBD development (Frias *et al.*, 1991), it was further investigated whether fungal sensing of carbon starvation could trigger the expression of fungal genes associated with the necrotrophic phase and the killing of infected tissues.

### Carbon starvation is a signalling component controlling the duration of the biotrophic phase of WBD

**Fig. 3.**
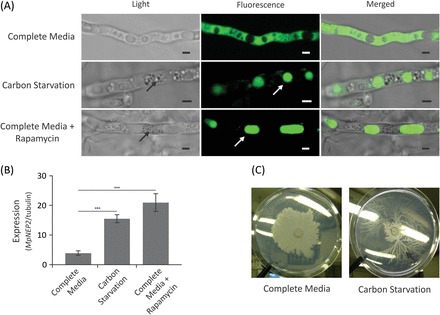
*MpNEP2* is up-regulated under autophagic conditions triggered by carbon starvation *in vitro*. (A) Light, fluorescence, and merged microscopy images of a transgenic *M. perniciosa* strain expressing the autophagy reporter *MpATG8-GFP*. In complete medium (upper panel), hyphae show uniform cytoplasmic distribution of fluorescent-tagged *ATG8* protein. In carbon starvation medium (middle panel) or in complete medium with rapamycin (lower panel), dense cytoplasmic granules (black arrows) and accumulation of protein into autophagic round vesicles are evident (white arrows). Scale bars=2 μm. (B) Relative *MpNEP2* gene expression under carbon starvation *in vitro* and in the presence of carbon with rapamycin. Data points represent a mean value of three biological replicates with calculated standard deviations (unpaired Student’s *t*-test; ***P<0.0005). (C) *M. perniciosa* grows as dense colonies in complete defined medium *in vitro*, while under carbon starvation growth is stunted, with the occasional appearance of fast-growing sectors showing hyaline hyphae with pronounced branching (black arrow). (This figure is available in colour at *JXB* online.)

It was previously shown that initiation of the necrotrophic phase of WBD *in vivo* is marked by the up-regulation of the fungal gene coding for an NEP-like effector of plant necrosis, *MpNEP2*, implicating its activity in the necrosis of infected cacao (Zaparoli *et al.*, 2011). Therefore, a test was conducted to determine if fungal carbon starvation could be part of the signal that triggers the expression of *MpNEP2*. The drug rapamycin and a transgenic *M. perniciosa* strain expressing a *GFP*-tagged autophagy-related protein (*MpATG8-GFP*) was first used to probe for the ability to set up a starvation condition in solid medium *in vitro*. Briefly, rapamycin target inactivates fungal TOR (target of rapamycin) kinases, triggering a traceable autophagic response that is characteristic of starvation even under the presence of an adequate amount of nutrients (Cutler *et al.*, 2001; Rohde and Cardenas, 2004). Transgenic lines grown in complete medium showed an even cytoplasmic pattern of ATG8–GFP protein localization, characteristic of non-autophagic fungal cells (Fig. 3A). Hyphae grown under carbon starvation accumulated cytoplasmic granules, and tagged ATG8 protein was found in typical round vesicles, presumably the autophagosomes of *M. perniciosa*. Finally, hyphae grown in complete medium containing rapamycin displayed a pattern similar to that verified in carbon-starved cultures (Fig. 3A), demonstrating the ability of rapamycin to bypass nutrient availability signals, triggering an autophagic response in *M. perniciosa* even when plenty of carbon is available. It was found that *MpNEP2* expression levels were significantly higher in both carbon-starved and rapamycin-treated cultures (Fig. 3B), suggesting that starvation up-regulates *MpNEP2* under these conditions *in vitro*. In addition, cultures transferred into starvation medium presented a peculiar phenotype of slow growth followed by the development of faster growing sectors formed by very thin mycelia with marked hyphal branching (Fig. 3C), a typical characteristic of the invasive growth observed in infected plant tissues after phase transition (Frias *et al.*, 1991).

**Fig. 4.**
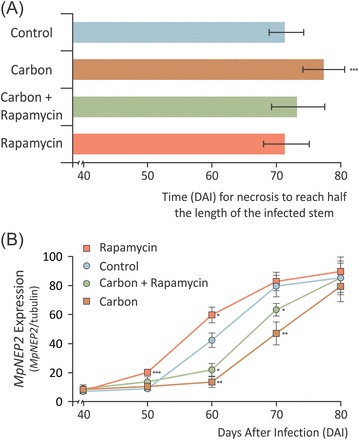
Manipulation of apoplastic fluid carbon availability alters *MpNEP2* expression and the necrosis rate of infected tissues. (A) Time (in days ater infection) that necrotic symptoms took to cover 50% of the length of infected stems after infiltrations with solutions containing carbon (sucrose, glucose, and fructose at 10mM each), carbon with 400ng ml^–1^ rapamycin, and rapamycin only. (B) *MpNEP2* gene expression assayed at 10 d intervals after infiltration of infected stems. Data points represent the mean of four biological replicates with calculated standard deviations (unpaired Student’s *t*-test; *P<0.05; **P<0.005; ***P<0.0005). (This figure is available in colour at *JXB* online.)

Next, advantage was taken of the extracellular confinement of *M. perniciosa* in green brooms to perform a petiole feeding assay (Lin *et al*., 2010, 2011) to manipulate apoplastic fluid sugar availability artificially and test if the fungal sensing of carbon starvation could be linked to *MpNEP2* expression and plant necrosis *in vivo*. Infiltration of a carbon solution on 40 DAI plants led to significant maintenance of higher carbon levels in the apoplastic fluid of infected tissues (Supplementary Fig. S4 at *JXB* online). This treatment led to a slowing down of the later necrotic process when compared with untreated controls (Fig. 4A), also followed by delayed *MpNEP2* up-regulation (Fig. 4B). This result indicates that the lack of soluble carbon in the apoplast is probably perceived by the infecting fungus and used as a cue to the time-opportune expression of *MpNEP2*.

In order to account for the possibility that the observed delay of plant necrosis was due to an increase in the survival of plant cells fed with carbon and thus not related to a fungal response to starvation, rapamycin was also included in the infiltrations. Because plant TOR kinases are reported to be insensitive to the drug (Menand *et al.*, 2002; Robaglia *et al.*, 2012), presumably rapamycin only inactivates fungal TOR kinase. This ‘differential targeting’ due to drug sensitivity should be able to rescue only the pathogen-related effects observed upon artificially increased sugar availability by infiltration. Rapamycin infiltration on healthy plants did not affect development nor did it induce any detectable symptoms (data not shown). Infected plants infiltrated with a carbon solution containing rapamycin presented a necrosis progression more similar to that verified in controls (Fig. 4A), showing that rapamycin is able to rescue the delayed necrosis phenotype of carbon-infiltrated plants. However, the timing of *MpNEP2* expression was only partially rescued (Fig. 4B), suggesting that other factors in addition to a response to carbon starvation may also be part of the signalling leading to the up-regulation of this effector *in vivo*. Accordingly, the infiltration of rapamycin alone did not induce significant acceleration of necrotic symptoms (Fig. 4A), and it led to only a slightly earlier up-regulation of *MpNEP2* (Fig. 4B). These results show that although *MpNEP2* up-regulation is partially associated with the the sensing of carbon starvation at the apoplastic fluid, it is probably not the main cause of necrosis in infected tissues. This suggests that either other unknown WBD effectors might be taking part in the biotrophic to necrotrophic transition upon fungal carbon starvation or that additional key processes involving the physiology of the host might also be at play. Because substantial carbon depletion in the apoplastic fluid of infected plants was found, it was decided to investigate further whether WBD-related plant carbon starvation could lead to an early commitment of infected tissues to the necrotrophic phase.

### WBD leads to a spatial pattern of intracellular sugar accumulation, photosynthesis decline, and up-regulation of carbon starvation markers within infected cacao tissues

The mature broom stage (30–40 DAI) precedes the appearance of the first signs of plant tissue death. This stage was characterized by the cessation of growth, with all apoplastic carbohydrate concentrations reaching the lowest levels measured during the experiment (Fig. 1B, C). This suggests that infected cacao tissues might have a compromised carbon metabolism and thus are unable to provide further the sugars to be released in the apoplast at this stage of WBD development. Infected plant tissues have long been regarded as having down-regulated photosynthesis (Orchard and Hardwick, 1988; Scarpari *et al.*, 2005), a condition known to set a scene of physiological carbon depletion leading to starvation and senescence in plants (Brouwer *et al*., 2013). To test if infected cacao tissues could be subject to carbon starvation, the expression of gene markers of acute carbon starvation in plants was assessed and photosynthesis and the main intracellular carbon pools were measured in the first fully expanded leaf at both the base and the top of control and infected stems.

**Fig. 5.**
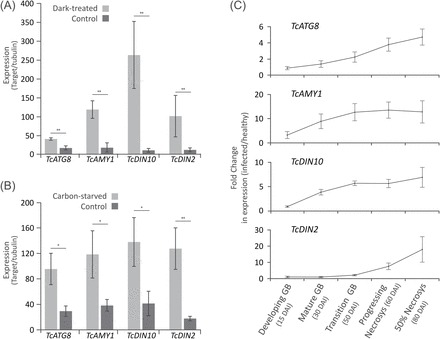
Cacao orthologues of markers of plant carbon starvation, *TcATG8i* (autophagy-related protein 8i), *TcAMY1* (α-amylase), *TcDIN10* (raffinose galactosyltransferase), and *TcDIN2* (β-glucosidase) are up-regulated upon carbon starvation in both tissues and cells as well as during WBD development. (A) Expression of carbon starvation markers in cacao leaves upon whole-plant dark treatment for 7 d. (B) Expression of marker genes in cacao cell suspension cultures without sucrose for 48h. (C) Expression of marker genes in whole infected tissues collected at key stages of WBD development, spanning from green broom stages (GB) to early necrosis. Data points represent the mean value of three biological replicates with calculated standard deviations (unpaired Student’s *t*-test; *P<0.05; **P<0.005; ***P<0.0005).

Cacao orthologues of previously characterized expression markers of carbon starvation in plants (Fujiki *et al.* 2000; Contento *et al.*, 2004; Rose *et al.*, 2006) were identified based on sequence similarity (Supplementary Table S1 at *JXB* online), and functionally validated in cacao tissues and cell suspensions cultures. qRT-PCR analysis showed an overall trend of up-regulation in the leaves of cacao plants incubated in the dark for 1 week (Fig. 5A) as well as in cacao cell culture without sucrose for 24h (Fig. 5B), confirming that the selected genes are responsive to standard carbon starvation conditions in plants. Up-regulation was also evident in infected cacao tissues collected during selected time stages of WBD development; however, distinct patterns could be identified between markers. While transcripts of the gene *TcATG8i* showed a relatively gradual increase during the stages, *TcAMY1* and *TcDIN10* appeared as early responsive markers, showing up-regulation at 30 DAI (Fig. 5C). In contrast, *TcDIN2* appeared as a late responsive marker, only being up-regulated at much later stages, at the spreading of infected tissue necrosis (Fig. 5C).

**Fig. 6.**
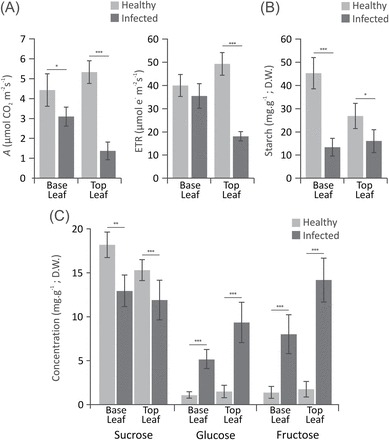
A spatial pattern of photosynthesis down-regulation and soluble sugar accumulation in leaves of mature green brooms. (A) Mean photosynthesis (*A*) and electron transport rate (ETR) levels in the first (older, at the base) and last (younger, at the top) fully expanded leaves of mature green brooms (30 DAI). (B) Mean levels of starch, and (C) sucrose, glucose, and fructose measured in the same leaves assayed for photosynthesis. Data points represent the mean value of eight biological replicates with calculated standard deviation (unpaired Student’s *t*-test; *P<0.05; **P<0.005; ***P<0.0005).

Photosynthesis (*A*) and electron transport rate (ETR) in the leaves at the top of 40 DAI stems were significantly reduced in infected plants when compared with leaves in the same stage and position of healthy controls (Fig. 6A). Although also affected, older leaves at the base had levels more similar to those of corresponding healthy controls, suggesting a spatial symptomatic gradient. Starch levels were also found to be reduced in infected leaves, but no significant positional differences were found (Fig. 6B). Surprisingly, while intracellular leaf sucrose levels are only slightly decreased, a marked accumulation of intracellular glucose and fructose occurs in the leaves of infected tissues. Moreover, leaf hexose accumulation appeared to occur to a greater extent in the top infected leaves (Fig. 6C), following the same pattern observed for the photosynthetic decline.

**Fig. 7.**
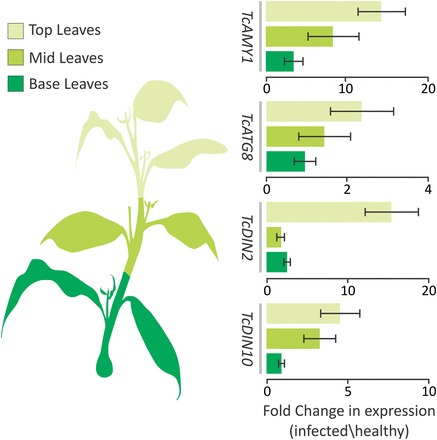
Cocoa orthologues of markers of plant carbon starvation are up-regulated following a spatial pattern in mature brooms. Fold increase of marker genes assayed at three different positions (shading) within mature brooms, showing either a gradual increase towards the top (early responsive genes *TcATG8*, *TcAMY1*, and *TcDIN10*), or specific up-regulation at the top leaves (late responsive gene *TcDIN10*). Data points represent the mean value of three biological replicates with calculated standard deviations. (This figure is available in colour at *JXB* online.)

In an attempt to correlate WBD-induced up-regulation of plant carbon starvation markers with the observed pattern of photosynthesis decline and intracellular hexose accumulation, their expression was evaluated in the same collected material used for photosynthesis and leaf carbon assays. Strikingly, it was found that the pattern of marker up-regulation followed the same basipetal gradient as observed for sugar accumulation and photosynthesis decay (Fig. 7), suggesting a direct correlation. Also, the patterns of early and late responsiveness that were evident at different developmental time stages (Fig. 5C) appeared also to occur within the vertical axis of shoots prior to phase transition. While the genes *TcAMY1*, *TcATG8i*, and *TcDIN10* showed a gradual increase in expression along the vertical axis of the infected shoot, the late responsive marker *TcDIN2* appears to be up-regulated only in the top leaves (Fig. 7). Taken together, the data suggest that physiological symptoms of WBD include dramatic changes in the primary carbon metabolism of cacao. Moreover, these changes appear to correlate well with the expression of genes involved in carbon starvation, occurring in a spatiotemporal gradient within the vertical axis of mature brooms.

## Discussion

### Apoplastic soluble sugars in the rhythmic growth of cacao and WBD development

Aiming for the characterization of the carbohydrates available for *M. perniciosa* in the apoplastic fluid of cacao, a concentration pattern that matched the periodic growth of healthy cacao seedlings was unexpectedly found (Fig. 1A, C; Supplementary Fig. S3 at *JXB* online). Cacao is known to have a periodic, flush-type growth, and early investigations (Greathouse *et al.*, 1971) showed exactly the same time frame of 30 d between early flushes that were verified in the present experiments (Fig. 1A). It was shown that cacao apoplastic fluid has considerably more carbohydra tes when the apical meristem is actively growing, precisely the only known vegetative tissues that are susceptible to WBD. In infected plants, levels of hexoses in the apoplastic fluid increased in parallel with symptom development and the decrease of sucrose levels (Fig. 1C, filled circles), correlating with the up-regulation of fungal and plant extracellular invertases during most of the green broom phase (Fig. 2A–C). The induction of cw-invertase activity has been reported as a feature common to many plant–pathogen interactions (Roitsch *et al.*, 2003). It often induces infected tissues to behave as carbon sinks, providing the pathogen with increased carbon resources (Berger *et al.*, 2007); however, the array of mechanisms used to manipulate host metabolism are far less understood. Infected cacao tissues have typical symptoms of hormone imbalance, notably involving auxin and cytokinin (Melnick *et al.*, 2012), and *M. perniciosa* has been shown both to possess the genetic pathways and to produce plant hormones *in vitro* (Kilaru *et al.*, 2007; Mondego *et al.*, 2008). Cw-invertases are known to be up-regulated in response to auxins and cytokinins (Roitsch *et al.*, 2003), and it is possible that a similar mechanism might be operating during the biotrophic phase of WBD. Recently, new evidence has emerged to support a major role of sugar availability as a determinant of apical dominance and bud dormancy break in plants (Mason *et al.*, 2014). It is noteworthy that hexose concentrations in the apoplastic fluid during the episodes of periodic growth of healthy plants are comparable with the abnormal levels sustained during the proliferative phase of developing green brooms (Fig. 1C). In addition to artificially sustaining sink metabolism in infected tissues, increased cw-invertase activity and the consequent higher levels of hexoses may also act additively with plant hormones as proliferative signals leading to the characteristic meristem hyperactivity, and the loss of both apical dominance and periodicity of growth in developing green brooms.

### Fungal carbon starvation at the cacao apoplast as a cue for the opportune expression of fungal effectors of plant necrosis

It was demonstrated that a decrease in the concentration of soluble carbohydrates occurs in the apoplastic fluid of mature green brooms (Fig. 1C), suggesting that *M. perniciosa* could be subject to a carbon starvation microenvironment immediately prior to the death of infected tissues. Starvation conditions have long been reported to induce fungal pathogenicity factors (Talbot *et al.*, 1997), and it has been shown here *in vitro* that the fungal NLP-like effector of plant necrosis, *MpNEP2*, is integrated downstream of a carbon starvation, autophagic response network (Fig. 3). Artificially increased carbohydrate concentrations in the apoplastic fluid of mature brooms led to a delay of plant necrosis and *MpNEP2* expression (Fig. 4), suggesting that carbon starvation at the apoplast is a significant signalling component ruling the timing of these events in WBD. However, the extent of the contribution of *MpNEP2* up-regulation in affecting the timing of necrosis was difficult to quantify. Although rapamycin was able to rescue the delayed necrosis phenotype observed in carbon-only infiltrations, the earlier *MpNEP2* expression observed in this treatment did not induce earlier necrosis (Fig. 4). This suggests that, instead of acting as the central causal agent of the death of infected tissues as previously hypothesized (Garcia *et al.*, 2007; Zaparoli *et al.*, 2011), this effector can rather be playing a synergic role to a previously set, ongoing plant cell death pathway.

### Patterns of intracellular hexose accumulation in infected stems suggest an early sugar-induced senescence of infected tissues

Exhaustion of apoplastic soluble sugars coincides with the cessation of growth and the beginning of the mature broom phase (Fig. 1B; Supplementary Fig. S3 at *JXB* online). This suggests that at this stage the infected tissues are unable to provide further the sugars to fuel the intense proliferative growth of green brooms, implicating a condition of carbon starvation. Further investigation of the physiological state of mature brooms revealed a spatial decline in photosynthesis that matched a pattern of intracellular hexose accumulation (Fig. 6A, C). Interestingly, vc-invertase appears more active in infected plants at this stage (Fig. 2A), providing a plausible cause for the higher intracellular sugar in infected tissues. Photosynthesis is known to be feedback mechanism inhibited by sugars (Paul and Pellny, 2003; Rolland *et al.*, 2006), suggesting a cause–effect relationship between hexose accumulation and photosynthesis down-regulation in mature brooms. Evaluation of the expression of starvation markers in the same material used for the photosynthesis and intracellular carbohydrate assays revealed a strikingly similar pattern (Fig. 7). Insightfully, the gene *TcDIN2*, which appeared as a late responsive marker in the different time stages of WBD (Fig. 5C), was found to be pronouncedly up-regulated only in the top leaves within the same stage (Fig. 7), suggesting that the distal parts of mature brooms are more advanced in the physiological symptoms related to the death of infected tissues. In fact, necrotic symptoms are known to be initiated distally, at the tips of the young, developmentally arrested leaves, moving on to the apical meristem and eventually all the infected stem (Purdy and Schmidt, 1996; Scarpari *et al.*, 2005; Meinhardt *et al.*, 2008). This temporal/spatial progression is remarkably similar to the pattern observed here for the reduction in photosynthesis and sugar accumulation, and up-regulation of plant starvation markers.

Although the present evidence suggests an appealing scenario where plant starvation triggers the senescence of infected tissues (Fig. 5C), the data regarding higher hexose content in leaves of mature brooms are seemingly contradictory (Fig. 6C). Cacao orthologues of known plant starvation markers were characterized and validated (Fig. 5A, B); however, the distinction between physiological causes leading to the senescence of plant cells using conventional markers is difficult because of shared, redundant response pathways (Gepstein, 2004). Accordingly, in a study addressing these differences using a whole-transcriptome approach, *Arabidopsis* orthologues of the markers used here to probe for cacao carbon starvation appeared to be up-regulated to different extents upon different types of senescence (Buchanan-Wollaston *et al.*, 2005). This shows that the selected markers used here are also likely to be part of the shared expression responses that occur during senescence in general, highlighting the importance of a multifaceted approach to distinguish between the different initial triggers of this process (Guo *et al.*, 2004; Breeze *et al.*, 2011; Trivellini *et al.*, 2012).

Nevertheless, the higher expression of the late responsive marker *TcDIN2* in tissues with higher intracellular sugar content (Figs 6, 7) suggests that sugar accumulation is highest in the tissues that are about to show symptoms of senescence. Sugar accumulation and low photosynthesis are among the proposed signals to regulate senescence in plants (Wingler *et al.*, 2009), and there is substantial debate as to whether the main causes are sugar starvation or sugar accumulation (Doorn, 2004; Rolland *et al.*, 2006; Wingler and Roitsch, 2008). It is noteworthy that in the tomato–*Xanthomonas campestris* hemibiotrophic interaction, invertase activity was directly implicated in symptom development, sugar accumulation, down-regulation of photosynthesis, and up-regulation of senescence-associated genes, suggesting that it leads to the senescence of infected tissues through restriction of carbon export and sugar accumulation (Kocal *et al.*, 2008). Thus it is believed that the present results better fit in a similar physiological scenario taking place during the development of WBD in cacao, and possibly indicating that the regulation of disease development through the disturbance to the patterns of host carbon metabolism might also be exploited by other plant pathogens.

In the particular case of WBD in cacao, sugar accumulation can explain down-regulation of photosynthesis, the up-regulation of starvation/senescence-related genes, and finally the onset of infected tissue senescence. However, the causes for the spatial–temporal dynamics depicted in the present data are unclear; however, future research effort into characterizing whole transcriptomic and metabolomic changes might provide a well defined picture of these dynamics. Finally, based on the present results, it is possible to put forward a hypothetical model of how cacao carbon physiology integrates in the biology of WBD development. Apoplast-dwelling hemibiotroph pathogens have to deal with the challenge of obtaining food while avoiding plant defences and the premature killing of infected tissues. Likewise, *M. perniciosa* cannot biotrophically access the nutrients inside cacao cells, and it relies on the later death of infected tissues to develop its reproductive structures, release spores, and complete its life cycle. During senescence, nitrogen and carbohydrates that are immobilized for the normal functioning of plant cells are converted into high valuable resources that are optimal for efficient remobilization and rapid utilization in storage and further plant growth (Lim *et al.*, 2007). By coupling the expression of *MpNEP2* to the onset of senescence using carbon starvation as a cue perceived at the apoplastic fluid, extracellular *M. perniciosa* hyphae can have early access to this valuable resource otherwise enclosed inside senescing plant cells. Moreover, opportune speeding up of infected tissue necrosis by *MpNEP2* activity might also prevent two important late features of plant senescence: remobilization and abscission. Accordingly, an intriguing characteristic of WBD in cacao is that dead infected tissues are preferentially kept attached to cacao trees for long periods of time (Purdy and Schmidt, 1996; Scarpari *et al.*, 2005; Meinhardt *et al.*, 2006). This can be advantageous to the pathogen by first allowing preferential access to the resources enclosed in dead tissues and thus not having to compete with other microorganisms that inhabit the ground litter of the forest. Secondly, it may also enhance the dispersal of spores and increase the chances of infecting nearby actively growing meristems of cacao trees, possibly completing the disease life cycle in a more efficient manner.

## Supplementary data

Supplementary data are available at *JXB* online.

Figure S1. Adapted Scholander pressure bomb to extract apoplastic fluids from cocoa shoots.

Figure S2. Validation of the pressure dehydration protocol for the extraction of apoplastic fluid of cocoa tissues.

Figure S3. Weight of control and infected tissues collected during the time course experiment reflects the differences between periodic growth and proliferative growth in healthy and infected tissues.

Figure S4, Carbohydrate concentrations in the apoplastic fluid of mature brooms infiltrated for 7 d with a water solution containing sucrose, glucose, and fructose, or water alone.

Figure S5. Outline of the cloned *EGFP-MpATG8* autophagy monitoring and hygromycin selection cassettes for *M. perniciosa* transformation.

Table S1. Oligonucleotide primers used in this study.

Supplementary Data
