# Supplementary material for: Apoplastic and intracellular plant sugars regulate developmental transitions in witches’ broom disease of cacao
Source: J Exp Bot. 2014 Dec 24;66(5):1325–37. doi: 10.1093/jxb/eru485 (PMC4339597; doi:10.1093/jxb/eru485)
Supplement: Supplementary Data [file supp_66_5_1325__index.html]

Apoplastic and intracellular plant sugars regulate developmental transitions in witches’ broom disease of cacao — Apoplastic and intracellular plant sugars regulate developmental transitions in witches’ broom disease of cacao — Supplementary Data 

# Apoplastic and intracellular plant sugars regulate developmental transitions in witches’ broom disease of cacao

## Supplementary Data

Data files

**Files in this Data Supplement:**

- Supplementary Data - Supplementary Data
