# Supplementary material for: Apoplastic and intracellular plant sugars regulate developmental transitions in witches’ broom disease of cacao
Source: J Exp Bot. 2014 Dec 24;66(5):1325–37. doi: 10.1093/jxb/eru485 (PMC4339597; doi:10.1093/jxb/eru485)
Supplement: Supplementary Data [file supp_eru485_jexbot131029_file001.pdf]

Supplementary Data

Apoplastic fluid extraction and validation

In order to extract apoplastic fluid (AF) from whole infected cacao tissues of seedlings, we chose a pressure-dehydration procedure with the use of a Scholander bomb (Fig. S1) (Scholander et al. 1965). Unlike the infiltration-centrifugation technique (Lohaus et al. 2001), this method allowed the extraction of AF from all the stages of Witches' Broom Disease (WBD). It has been also previously used to extract AF from the woody perennials, poplar and holm oak, in addition to whole tomato fruits and a few other plant species (Pechanova et al. 2010; Gabriel and Kesselmeier 1999; Ruan et al. 1995; Hartung et al. 1988; Jachetta et al. 1986; Cornish and Zeevaart 1985).

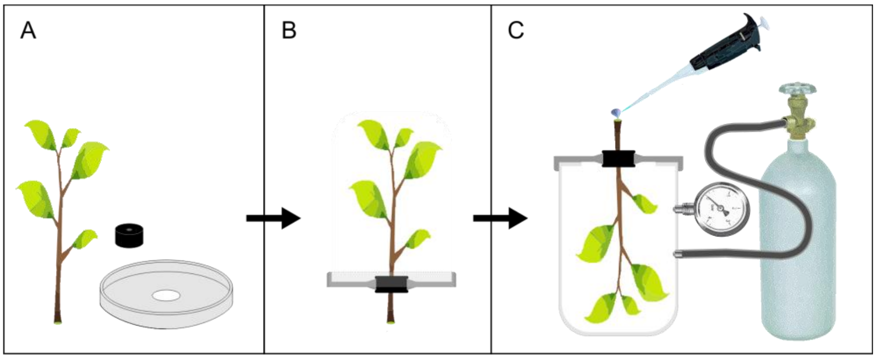

Supplemental Figure S1: Adapted Scholander pressure bomb to extract apoplastic fluids from cocoa shoots.

Using this system, we obtained larger volumes at higher pressures, but at expense of increased cell damage as assayed by the intracellular marker activity, Glucose 6-phosphate isomerase (*HPI*). However, the application of 20 Bar enabled the isolation of up to 50 µL.g^{-1} (fresh weight) of AF containing less than 0,2 percent of cell rupture and cytoplasmic contamination (Fig. S2A). Moreover, the amino acid profile is significantly different between AF and whole tissues extracts (Fig. S2), ruling out the possibility of extensive contamination of samples with membrane and cell wall-filtered low molecular weight metabolites.

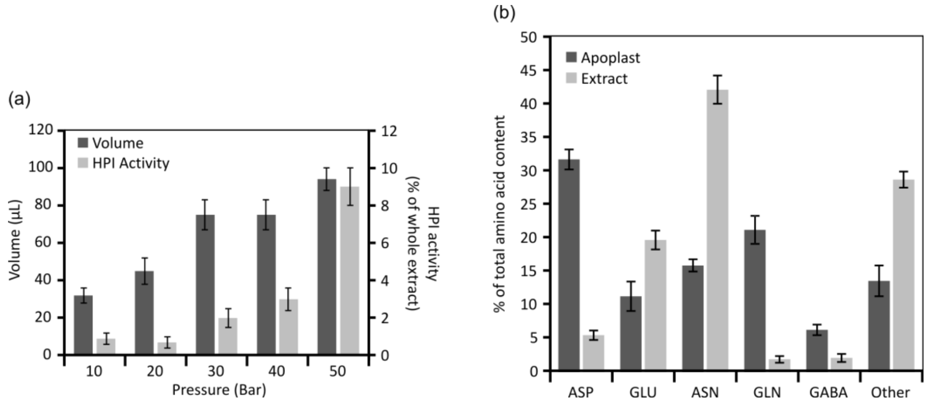

Supplemental Figure S2: Validation of the pressure dehydration protocol for the extraction of apoplastic fluid of cocoa tissues. (a) Apoplastic fluid volume and hexose-phosphate isomerase activity in function of the applied pressure on the extraction. (b) Amino acid profile of extracted apoplastic fluid and in the extract of whole cocoa tissues.

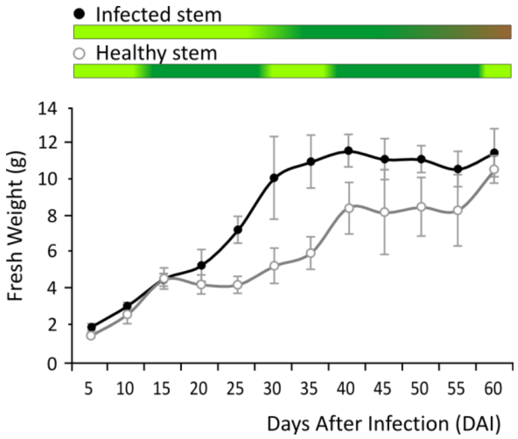

Supplementary Figure S3: weight of control (open circles) and infected (closed circles) tissues collected during the time-course experiment reflects the differences between periodic growth and proliferative growth in healthy and infected tissues (summarized in the upper color bars: light green reflects active flushing and leaf expansion, and dark green reflects leaf maturation).

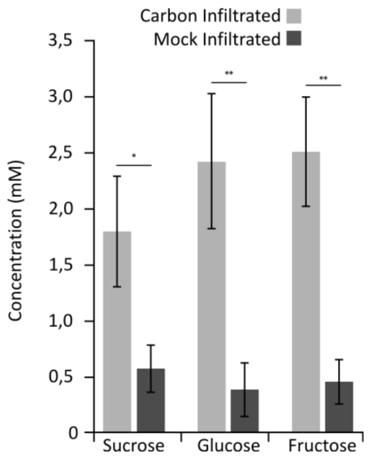

Supplementary Figure S4: Carbohydrate concentrations in the apoplastic fluid of mature brooms infiltrated for 7 days with a water solution containing sucrose, glucose and fructose, or water alone. Measures were conducted seven days after the end of the infiltration period.

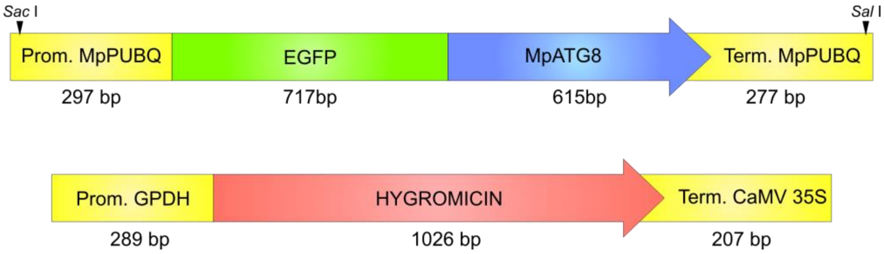

Supplementary Figure S5: outline of the cloned *EGFP-MpATG8* autophagy monitoring and hygromicin selection cassettes for *M. perniciosa* transformation.

Supplemental Table 1: oligonucleotide primers used in this study

Primer Name	Sequence (5'-3')	Target
Ppubq-FWD	TGATGTCAGGTTCATCCAAC	*M. perniciosa* polyubiquitin gene promoter
Ppubq-REV	CCTCGCCCTTGCTCATTCTGGAATATGGAGACGATG	
Tpubq-FWD	GCAGTGCTGTTGTAGTTGCC	*M. perniciosa* polyubiquitin gene terminator
Tpubq-REV	CAGACTGCATCTTCTACGTC	
MpATG8-FWD	ACAAGATGAGGTCCAAATTCAAGGATG	*M. perniciosa MpATG8* gene
MpATG8-REV	GGCAACTACAACAGCACTGCAATATCCATGAATCCGACGGTAGCT	
eGFP-FWD	CCAGAATGGTGACGAAGGGCGAGG	*EGFP* coding sequence from pBGgHg
eGFP-REV	CATCCTTGAATTTGGACCTCATCTTGTACAGCTCGTCCATG	
PpubqN1-FWD	AGAGCTCATAGCAACCGCTTCGATGG	Nested PCR of *EGFP-MpATG8* cassette with *Sac*I and *Sal*I sites
TpubqN2-REV	CTACGTCGACTAACGTGACCGCTCAGGTGG	
hph-FWD	AACAACTCTTCCTTTCAACCACAAGGAAGAAGCTTTAAGAGGTCC	PCR of hygromycin selection cassette from pBGgHg
Hph-REV	CGCTCAACAAGTGCAGCTTTCAGACCGTTAATAACACATTGCGGACG	
TcDIN2 FWD	TGATGCACCTAAGCATGG	qRT-PCR of markers of acute carbon starvation in *T. cacao*: beta-glucosidase (*DIN2* - XM_007014820.1); alpha amylase (*AMY* - XM_007017177.1); raffinose galactosyltransferase (*DIN10* - XM_007013477.1); autophagy-related protein 8 (*ATG8* - XM_007026030.1*)*
TcDIN2 REV	ATCCGAAGCAGTTGTTGG	
TcDIN10 FWD	GCTTATTCATCAGCAAAGCC	
TcDIN10 REV	AGCTGAACTTCACCAATCC	
TcAMY1 FWD	GAGAGGAAAGATGGAAGAGG	
TcAMY1 REV	CATCGCCACAAATGAAGG	
TcATG8 FWD	CAAGCTTGAACATCCTTTGG	
TcATG8 REV	CCTTCTCCACGATAACAGG	
TcINV1 FWD	GAGGAGCTTGATAGATCATTCC	qRT-PCR of *T. cacao* invertase
TcINV1 REV	GGCAGCGTTGTTAATTGC	
TcTUB FWD	TCCCAACAATGTGAAGTCC	qRT-PCR of *T. cacao* tubulin (endogenous control)
TcTUB REV	CATCTCCTGGATTGATGTCG	
MpINV1 FWD	TGCTGTGTCGTTCTTACC	qRT-PCR of *M. perniciosa* invertase gene
MpINV1 REV	TCAAAGGTATAGCCAGTGC	
MpNEP2 FWD	TTCTTGGGAAGGTATGGG	qRT-PCR of *M. perniciosa NLP*-like gene 2
MpNEP2 REV	GAGTCAATGAGCGAATCC	
MpTUB2 FWD	TGTCAACCTGGTTCCTTTCC	qRT-PCR of *M. perniciosa* tubulin (endogenous control)
MpTUB2 REV	GACAGCACGGTACTGTTGG	

Supplemental references:

Gabriel, R. & Kesselmeier, J., 1999. Apoplastic Solute Concentrations of Organic Acids and Mineral Nutrients in the Leaves of Several Fagaceae. Plant and Cell Physiology, 40(6), pp.604–612.

Hartung, W., Radin, J. & Hendrix, D., 1988. Abscisic Acid Movement into the Apoplastic Solution of Water-Stressed Cotton Leaves. Plant Physiology, 86(3), pp.908–913.

Jachetta, J., Appleby, A. & Boersma, L., 1986. Use of the pressure vessel to measure concentrations of solutes in apoplastic and membrane-filtered symplastic sap in sunflower leaves. Plant Physiology, 82(4), pp.995–999.

Lohaus, Gertrud et al., 2001. Is the infiltration-centrifugation technique appropriate for the isolation of apoplastic fluid? A critical evaluation with different plant species. Physiologia Plantarum, 111(4), pp.457–465.

Pechanova, O. et al., 2010. Apoplast proteome reveals that extracellular matrix contributes to multistress response in poplar. BMC genomics, 11(1), p.674.

Ruan, Y. et al., 1995. Non-Destructive Collection of Apoplast Fluid From Developing Tomato Fruit Using a Pressure Dehydration Procedure. Australian Journal of Plant Physiology, 22(5), pp.761–769.

Scholander, P. et al., 1965. Sap Pressure in Vascular Plants. Science, 148(3668), pp.339–346.
